# Complete Hematological Response Coupled with Molecular Response in Polycythemia Vera Treatment with Ropeginterferon Alfa-2b

**DOI:** 10.3390/life16081220

**Published:** 2026-07-23

**Authors:** Veronica Vecchio, Rossella Cacciola, Emma Cacciola

**Affiliations:** 1 Hematology Unit with BMT, A.O.U. Policlinico “G. Rodolico-San Marco”, 95123 Catania, Italy; 2 Department of General Surgery and Medical Surgical Specialities, University of Catania, 95131 Catania, Italy; 3 Hemostasis/Hematology Unit, A.O.U. Policlinico “G. Rodolico-San Marco”, 95123 Catania, Italy; rcacciol@unict.it (R.C.); ecacciol@unict.it (E.C.); 4 Department of Clinical and Experimental Medicine, University of Catania, 95131 Catania, Italy; 5 Department of Medical, Surgical Sciences and Advanced Technologies “G.F. Ingrassia”, University of Catania, 95131 Catania, Italy

**Keywords:** polycythemiavera, ropeginterferon alfa-2b, complete hematological response, durable CHR, JAK2V617F variant allele frequency

## Abstract

**Background:** Polycythemia vera (PV) is characterized by mutation JAK2 V617F, elevated blood counts, and risk of thrombosis. Ropeginterferon alfa-2b (ropeg) approved for PV at a low-starting dose and slow-titration schema with efficacy at 12 months with assessment visits every three months. This period might be at thrombotic risk resulting from inadequate control of hematological parameters and JAK2. The study was aimed at assessing its efficacy at less than 12 months with assessment visits every two weeks. **Methods:** Thirteen patients with Hydroxyurea (HU)/Intolerance (HU/I) high-risk PV and pruritus were treated with ropeg at a low-starting dose and slow-titration schema with assessment visits every two weeks. The primary endpoint was the complete hematological response (CHR) at less than 12 months. The secondary endpoints were the CHR rates, CHR dose, durable CHR, and reduction in JAK2V617F variant allele frequency (VAF). **Results:** The CHR of patients was 8 weeks in 30.7% (4/13), 16 weeks in 30.7% (4/13), and 24 weeks in 38.4% (5/13), respectively, with a mean time of 4.15 months. Durable CHR was at 20, 28, and 36 weeks, respectively, with a mean time of 6.55 months. JAK2 VAF continuously declined from 43.38% at baseline to 12.96% at durable CHR. Eleven patients (85%) achieved partial molecular response (PMR) and two (15.38%) complete molecular responses (CMRs) and all patients resolved the pruritus. No adverse event (AE) or thrombosis occurred. **Conclusions:** We report that ropeg is efficacious under the low-starting dose and slow-titration regimen in obtaining CHR and molecular response (MR) at less than 12 months.

## 1. Introduction

Polycythemia vera (PV) is a chronic myeloproliferative neoplasm characterized by substantial morbidity and mortality due to thrombotic events. Elevated red blood count (RBC), responsible for elevated hematocrit (HCT), white blood cell (WBC), and platelet (PLT) elevated counts increase the blood viscosity causing circulatory disorder and thrombotic risk [1]. The pantocytosis is driven by the JAK2V617F variant allele frequency (VAF) [1]. The JAK2 mutation is present in nearly all patients with PV [2] and its detection is a major criterion for PV diagnosis associated with the aberrant proliferation of hematopoietic cells [3]. Patients with PV are at risk of progression to secondary myelofibrosis or acute myeloid leukemia [4]. However, the main concern is an increased risk of thromboembolic events (TEs), with a 2.7- to 13.1-fold higher occurrence of arterial and venous thrombosis, respectively [5]. Arterial thromboses, including transient ischemic attack, ischemic stroke, myocardial infarction, and unstable angina, are more common than venous TEs, including deep venous thrombosis of the legs, pulmonary embolism, and venous thrombosis in atypical sites [6]. TEs may occur before or at the diagnosis of PV with a rate reducing overtime due to the effect of the therapy [6,7,8,9,10]. A history of thrombosis is the main predictor of reduced life expectancy in patients with PV [11] with a median overall survival of 5.1 years in patients suffering of a TE within 1 year of PV diagnosis [12]. Thrombosis was also associated with a doubled risk of mortality and accelerated transformation to myelofibrosis or acute leukemia [13]. Risk factors for TEs represent an important topic of investigation. Advanced age (≥60 years) and/or previous thrombosis are well-known predictors of frequent thrombosis [6,9,14,15,16] and classify the patients with PV into high risk whereas their absence classifies them into low risk [17]. Aspirin, periodic phlebotomies, and hematocrit ≤45% are required for reducing the risk of thrombosis in all patients [17]. Cytoreductive therapy is recommended for patients with high-risk PV [4,17]. However, advanced age and prior TEs provide only partial view of the overall thrombotic risk profile [2]. Recent evidence suggests that JAK2V617F mutation present in more than 90% of patients diagnosed with PV can impact the thrombotic risk contributing to hypercoagulability and has been associated with increased risk of thrombosis even in the absence of myeloid disorder [18,19]. The PROSPERO study (Trial registration number: NCT05548062, date of trial registration: 21 September 2022) focuses on high-risk PV patients with prior TE and receiving either hydroxyurea (HU) or ruxolitinib to identify factors contributing to thrombotic risk by collecting clinical, laboratory and treatment-related parameters [2]. The design of our research focuses on high-risk PV patients with advanced age and receiving ropeginterferon alfa2-b (ropeg) to identify factors contributing to thrombotic risk by collecting clinical, laboratory and treatment-related parameters. The short-term goal is to prevent thrombosis, by obtaining a rapid complete hematological response (CHR) associated with molecular response (MR); the long-term goal to prevent leukemia and myelofibrosis [2]. Ropeginterferon alfa2-b (ropeg) (Besremi) is a pegylated recombinant human interferon (IFN) approved for the treatment of PV with no symptomatic splenomegaly based on the PROUD/Continuation-PV studies [20]. Its prolonged half-life is related to the attachment of a polyethylene glycol (PEG) molecule to interferon alfa, allowing for a low-starting dose and slow-titration schema [21]. PV studies utilizing efficacy assessment visits every 3 months either with a low-starting dose and slow-titration schema or higher-starting dose and faster-titration schema obtaining CHR at 12 months [22,23]. We, utilizing efficacy assessment visits every two weeks with a low-starting dose and slow-titration schema, obtained CHR at 4.15 months. Nine dosages given every two weeks with an initiating dose of 100 μg followed by 50 μg intra-patient dose increments (100–150 first month, 200–250 s month, 300–350 third month, 400–450 fourth month, 500 fifth month) were explored for a total of 36 weeks. In total, 4/13 (30.7%) patients (Group A) achieved CHR at 8 weeks with a mean dose of 175 μg (range 100–300), 4/13 (30.7%) patients (Group B) at 16 weeks with a mean dose of 212 μg (range 100–300), and 5/13 (38.4%) patients (Group C) at 24 weeks with a mean dose of 280 μg (range 300–500) with a mean overall dose of 265.38 μg, significantly lower than mean doses of 540 μg or 500 μg reported in PV studies [20]. A durable CHR was obtained at a mean time of 6.55 months (28.46 weeks) as well as MR, differently from PROUD/Continuation-PV and PEGINVERA slow-titration studies obtaining durable CHR and MR at 12, 24 and 36 months and at 34 weeks, respectively, and Suo’s higher-titration study obtaining durable CHR at 36 and 52 weeks [20,23]. Our findings show that a closer intra-patient visit schema might have the potential to better measure the CHR time avoiding high doses and toxicities.

## 2. Materials and Methods

We performed a retrospective study using 13 patients’ data diagnosed with PV according to WHO 2016/ICC 2022 followed between January 2024 and January 2026 acquired from Hematology Unit and Bone Marrow Transplantation Unit of Azienda Ospedaliera Policlinico G. Rodolico San Marco of Catania. Participants met the following inclusion criteria to take part in this study: (1) signed informed consent; (2) age ≥18 years; (3) high-risk stratification according to ELN classification [4]; (4) normal spleen size; (5) at least one symptom (pruritus); and (6) treatment with ropeginterferon alfa-2b. The main objective was the evaluation of the CHR time. The sex distribution unusual of patients (11 males, 2 females) is referred to Deadmond MA et al. [24]. The mean age of the patients was 70 years (range 57–85 years). All patients had high-risk PV and had Hydroxyurea (HU)/Intolerance (HU/I) defined, according to ELN criteria [25], after about 100 days of therapy, and the presence of gastrointestinal symptoms and HCT at beginning of interferon alfa of more than 50% was due to suspension of HU and refusal of erythrocyte apharesis. All patients had JAK2V617F mutation (mean VAF 43.38%) and were treated with ropeginterferon alfa-2b at low-starting dose of 100 μg followed by slow-titrations of 150, 200, 250, 300, 350, 400, 450, and 500 μg doses subcutaneously every two weeks. The mean dose was 265.38 μg. All patients had pruritus with a MPN-SAF TSS (Myeloproliferative Neoplasm Symptom Assessment Total Symptom Score) score of 5.46 [26] (Table 1). The primary endpoint was the CHR at less than 52 weeks, defined as HCT < 45%, leukocyte count < 10 × 10^9^/L, and PLT count ≤ 400 × 10^9^/L, and resolution of pruritus, utilizing screening visits every two weeks. The secondary endpoints included CHR rates, CHR dose, durable CHR, and reduction in JAK2 V6717F VAF.

All analyses were performed using IBM SPSS statistics 25.0. Obtained results were expressed using mean value and standard deviation. Correlations were performed using Pearson and Spearman tests. Two-tailed *p* < 0.05 was considered statistically significant.

Ethical Considerations

The study was conducted in according with the Declaration of Helsinki. The retrospective study involving humans was conducted according to a protocol approved by the local ethical committee (Comitato Etico Catania 1, Azienda Ospedaliero Universitaria Policlinico-Vittorio Emanuele, identification code #34/2013/VE). Written informed consent was obtained from all participating patients in this study.

## 3. Results

Thirteen patients with PV were enrolled in this study. The mean age at enrollment was 70 years. All patients had previously received and had become intolerant to HU treatment [25]. No patients had previously suffered thrombosis. Differently from the PV studies that planned efficacy assessment visits every three months obtaining CHR at 12 months [22,23], we planned efficacy assessment visits every two weeks, obtaining CHR at a mean time of 16.53 weeks (95% CI: 8–24) equal to 3.80 months. The mean time of CHR was further assessed as the number of months between the initiation of treatment and the time to CHR and was 4.15 months (95% CI: 2–6).

The CHR rates, based on low-starting-dose and slow-titration schema, at 8, 16, and 24 weeks of treatment were 30.7% (4/13 group A), 30.7% (4/13 group B), and 38.4% (5/13 group C), respectively, with an overall CHR rate of 99.9%. In PROUD-PV study, based on similar dose initiation and titration, the CHR rate was 21% at 12 months and 43% after 1 year [3,5]. In a PVstudy, based on higher dose initiation and higher-titration schema, the CHR rate was 61.2% at 24 weeks and 71.4% at 52 weeks [23,27].

In our study, 4/13 patients (Group A) achieved CHR at 8 weeks with a mean dose of 175 μg (range 100–300), 4/13 patients (Group B) at 16 weeks with a mean dose of 212 μg (range 100–300), and 5/13 patients (Group C) at 24 weeks with a mean dose of 280 μg (range 300–500) with an overall mean dose of 265.38 μg, significantly lower than mean doses of 540 μg or 500 μg reported in PV studies [20]. HCT values are extremely high 56, 56, and 64% and this indicates the suspension of HU. We started the patient with a HCT of 64% with interferonalfa without prior phlebotomy to lower the HCT because the patient refused it. Differences among the three groups in terms of when they reached CHR could be due to accompanying lower hematological parameters (Table 2).

The durable CHR, defined as CHR lasting for at least 12 weeks [28], was observed in all three groups of patients from week 8 to 20 (30.7%), week 16 to 28 (30.7%), and week 24 to 36 (38.4%), respectively, with a durable CHR from week 20 to 36 of 99.9% with a mean time of 28.46 weeks (6.55 months) (Table 2). This rate of durable CHR was higher than previously observed in the PV studies with ropeginterferon alfa-2b at the slow-titration schema from week 36 to 52 and at higher-titration schema from week 24 to 36 with values of 27.6% and 43.7%, respectively [23,29].

In 13 patients, the JAK2V617F VAF and pruritus continuously declined from 43.38% at baseline to 12.96% with a decrease of 72.09% as well as pruritus from 5.46 score at baseline to 0 score at a mean time of 28.46 weeks (6.55 months) of the durable CHR and a significant correlation was found between JAK2 VAF and pruritus (Table 3). No information about spleen size during therapy is given because the patients had normal spleen size.

Based on the 2009 and 2013 criteria [28,30], 11 patients (84.61%) with a baseline mean VAF of 43.63% achieved PMR, defined as a reduction ≥50% in patients with VAF greater 10% or 20%, of 15.32% at a mean time of 28 weeks (6.44 months) of durable CHR with a decrease of 65.11%. Two patients (15.38%) with a baseline mean VAF of 25.50% achieved CMR, defined as a undetectable VAF level, of 0.135% at a mean time of 32 weeks (7.36 months) of durable CHR with a decrease of 99.47%. Differently, in a PV study two patients showed a undetectable VAF level at week 52 [23]. These results show that close visits rather than later visits can more effectively capture a rapid CHR controlling the thrombotic risk.

## 4. Discussion

Our study demonstrates that the 13 studied patients treated with Besremi as second-line treatment after HU on low-starting dose and slow-titration regimen without phlebotomy or erythrocyte apharesis attending assessment visits every two weeks show a rapid and favorable CHR differently from the 13 patients in Grasu’s study attending assessment visits every 2/3 months showing a delayed and unfavorable CHR [1].

**Figure 1:**
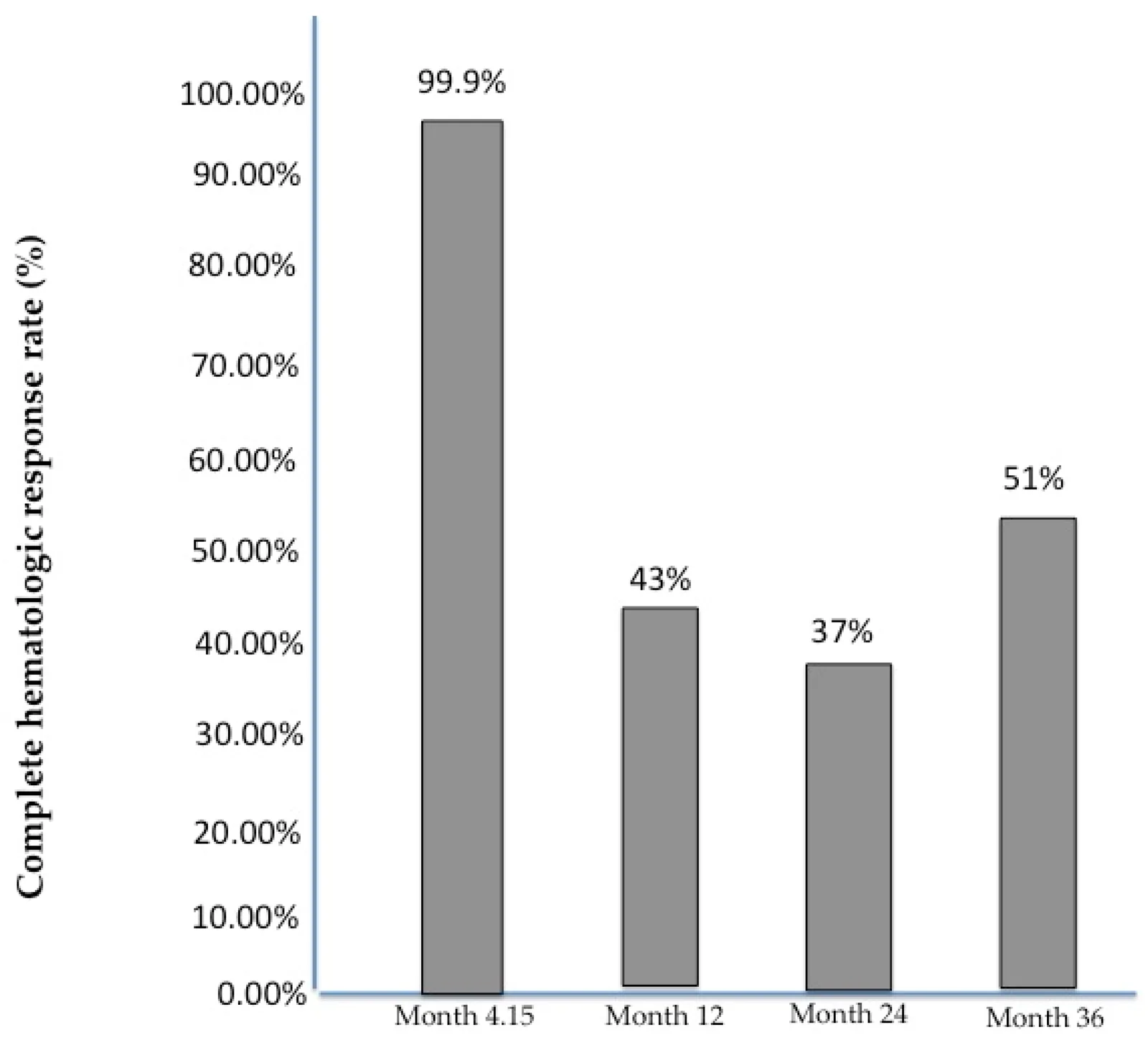
Complete hematological response (CHR) rates and time to CHR.

The mean time of CHR was 4.15 months significantly lower than previously reported either for slow-titration or higher-titration dosing schema that was 12 months [20,22,23]. The CHR rates were 99.9% within 24 weeks as compared to 43% and 51% at month 12 and 37% at month 24, and 51% at 36 months of treatment with the slow-titration schema in the PROUD-PV study and in a Japanese multicenter PVstudy and CONTINUATION-PV study, respectively, (Figure 1) and 71.4% observed following 52 weeks with the high-titration schema in the study by Suo et al. [22,23,29].

The mean dose at CHR was 265.38 μg, very much lower than the mean dose of 540 μg in the PEGINVERA study or 500 μg in the PROUD-PV study with a slow-titration schema and in a phase 2 Chinese study with a higher-titration schema [20,31].

In our study we obtained durable CHR, according to ELN 2009 and 2013 criteria, associated with a significant reduction in JAK2V617F VAF from 43.38% at baseline to 12.96% at 6.55 months. These results suggest that the therapy of the patients with PV at the slow intra-patient dose titration regimen can have a consistent efficacy on JAK2V617F malignant clone normalizing the hematological parameters, comprising HCT, WBC, and PLT counts, and controlling the thrombotic risk [32,33].

As opposed to control of thrombosis, the indication for treatment includes the control of symptoms such as the pruritus [34]. Pruritus is a PV-burden symptom, often exacerbated by contact with water [22,35]. A German large study on 441 patients with PV reported that 68% of the patients suffered from aquagenic pruritus [36]. Pruritus can be present before diagnosis of PV and includes different sensations such as burning, itching, stinging, or tickling and its severity is often unbearable [36]. Application of different treatments, including antihistamines, antidepressants, phototherapy, iron supplements and myelosuppressive drugs such as HU have poor effectiveness as well as non-drug medications, including avoidance of dry skin, temperature control of one’s environment and water used for bathing [37]. There is accumulating evidence of efficacy of the peg-IFN in alleviating the intractable pruritus [38]. In Lee’s et al. study 19 patients with HU/I low risk PV treated with ropeg showed modest improvement or worsening in pruritus [39]. In our study 13 patients with HU/I high-risk PV treated with ropeg showed improvement and overall resolution in pruritus.

The Myeloproliferative Disorders Research Consortium study (MPD-RC)-111 studied 50 HU/I high-risk PV patients with splenomegaly treated with PeGylated IFN alfa2a (PEG) showing CHR with resolution of splenomegaly in 11/50 (22%) at 12 months, although 27/50 (54%) of patients were receiving phlebotomy [40]. The PROUD-PV study studied 122 patients HU/I high-risk PV patients treated with ropeginterferon alfa-2b showing CHR with resolution of splenomegaly in 26 (21%) patients at 12 months [22]. Our study studied HU/I high-risk PV patients treated with ropeginterferon alfa-2b showing CHR with improvement in pruritus in 13/13 (99.9%) at 4.15 months and resolution at 6.55 months of the durable CHR and no patients received phlebotomy. Clinical and hematological efficacy of IFN-alfa-based therapy in myeloproliferative neoplasms has been reported since the 1980s; however, toxicity may led to rate of unintended discontinuation or interruption. Treatment is discontinued in the case of unresolved treatment-related toxicity or interrupted in the case of grade 3 or higher toxicity or dose reduction for grade 2 toxicity [5]. It is reported that the recombinant rIFN-alfa has a frequency of toxicity of ~10–20% [21]. Toxicity to rIFN-alfa includes flu-like syndrome, hepatic, endocrine, autoimmune, neurologic and psychiatric disorders [21]. The flu-like syndrome may develop during rIFN-alfa treatment and the patient may be supported with paracetamol. Abnormal liver test such as elevated alanine aminotransferase (ALAT) may occur during rIFN-alfa treatment. This elevation may be transient, reversible, and clinically insignificant. A 2x rise in ALAT and bilirubin does not preclude continuation of treatment but it is necessary to monitor monthly the ALAT levels. In some patients, the rIFN-alfa therapy may be paused until abnormal liver tests have normalized. Secondary hypothyroidism is not a contraindication since the hormonal replacement therapy is implemented. Hyperthyroidism is a contraindication for continuation of rIFN-alfa therapy. The presence of autoimmune disease, such as rheumatoid arthritis, or neurological disorder require a rheumatical or neurological consultation [21].

Side effects during treatment with rIFN-alfa are reported in Lee, Yacoub, and Gisslinger’s studies [22,39,40] with rates of 66.7%, 13.9%, and >10%, respectively. In particular, in Lee’s study the majority were of mild or moderate grade and included elevated liver enzymes and alopecia [39], and in the Yacoub’s study [40] the majority were of all grades and manageable and included diarrhea or fatigue, whereas in the Gisslinger’s study [22] the majority were grade 3 and grade 4 and included increased γ-glutamyltransferase and alanine aminotransferase [22]. In our study no patient had any adverse effects and the therapy was neither interrupted nor paused.

Both hematological parameters as well as JAK2V617F are risk factors for PV patients to develop thrombosis [23]. Therefore, it is desirable to reach the CHR and durable CHR as soon as possible.

The PROUD-PV study reports a decrease in JAK2V617F VAF from 41.9% at baseline to 30.7% at 52 weeks while the CONTINUATION-PV study reports a decrease in JAK2V617F VAF from 42.8% at baseline to 19.7% at 156 weeks [22]. The Myeloproliferative Disorders Research Consortium study (MPD-RC)-112 studied 36 high-risk PV patients treated with PeGylated IFN alfa2a (PEG) showing a reduction in the JAK2V67F VAF from 35% at baseline to 10.7% at 24 months, not associated with CHR [41]. We studied 13 high-risk PV patients treated with ropeginterferon alfa-2b showing a reduction in JAK2V617F VAF from 43.38% at baseline to 12.96% associated with the durable CHR.

In addition, Suo et al. report two patients with CMR. One patient had a baseline JAK2V617F VAF of 59.2% and the other had 4.7%. The JAK2V617F VAF was undetectable for both patients after 52 weeks of treatment [23]. We also report two patients with CMR. One patient had a baseline JAK2V617F VAF of 40% and the other had 11%. The JAK2V617F VAF was undetectable for both patients at 32 weeks of treatment. Based on 2009 and 2013 criteria, Suo et al. report PMR in 13 patients (28.3%) with a decrease in JAK2V617F VAF to less than 10% after 52 weeks of treatment [23]. Based on the 2009 and 2013 criteria, we report PMR in 11 patients (84.6%) with a decrease in JAK2 V617F VAF to 15% after 28 weeks (6.44 months).

It is reported that a JAK2V6717F allele burden of more than 20% increases the risk of thrombosis 7.4-fold [25,42]. In our study, we observed that the ropeginterferon alfa-2b at a dose of 265.38 μg reduces the JAK2V617F VAF at less than 20% (12.96%), resulting in an adequate control of risk of thrombosis. In addition, this finding could be consistent with the notion that ropeginterferon alfa-2b can have a selective effect on malignant clone differently from PEG-IFN alfa-2a [43,44,45,46]. There are limitations to this study, the greatest limitation being the overall small cohort size. It must be mentioned that patients lost to follow-up was the main reason. Additionally, the single-center design also constitutes a limitation. We performed a single-center design considering relevant advantages including simplicity, low costs, and a direct overview and control on over all aspects of the study. The retrospective nature of the research allows us to generate preliminary findings that can inform future and more extensive research. The lack of a control group/comparator arm is due to subjects who are similar overall but have different exposure to thrombosis. The short-term follow-up period requires further studies for a long-term follow-up period in order to confirm the findings of this study and the favorable safety profile in the long term.

## 5. Conclusions

The paper discusses an important issue in the management of PV patients, especially regarding optimal dosing and scheduling to reduce thrombosis while achieving rapid and sustained hematological and molecular remissions. Frequent bi-weekly visits yield interesting information on the kinetics of response. Earlier achievement of CHR (4.15 months) and a significant drop in the level of JAK2V617F mutant allele frequency at low dosages of the drug were observed than any previous research studies. These results should be validated via a large randomized trial.

## Figures and Tables

**Table 1:** Patients demographics and baseline characteristics.

Age (years), mean	70 (plus/minus 85/57)
Sex	Male 11 (85%) Female 2 (15%) [24]
PV diagnosis (months), mean	23 (plus/minus 48/12)
History of HU treatment	
Intolerance	13 (100%)
Total duration of HU treatment (days), median (min.–max.)	100.0 (90.00–120.00)
Besremi dose (μg), mean	265 (plus/minus 500/100)
Phlebotomy or Erythrocyte apharesis	refused
History of thrombosis	0
Spleen size (cm) *, mean	11.5 (plus/minus 12/10)
Pruritus (MPN-SAF TSS score), mean	5.46 (plus/minus 8/2)
JAK2V617F VAF (%), mean	43.38 (plus/minus 80/6)
Hematocrit (%), mean	55.61 (plus/minus 64/52)
Leukocytes (10^9^/L), mean	11.38 (plus/minus 21/6)
Platelets (10^9^/L), mean	484.07 (plus/minus 800/188)

* Spleen size measured by ultrasound.

**Table 2:** Statistical analysis of subgrouped patients (A,B,C) according to CHR and durable CHR time after treatment with Besremi.

	A	HCT * (52–56)	WBC * (6–12)	PLT * (255–719)		B	HCT * (52–56)	WBC * (8–21)	PLT * (272–799)		C	HCT * (53–64)	WBC * (7–12)	PLT * (188–714)
Baseline		53.0000	11.0000	430.0000	Baseline		55.0000	15.0000	542.5000	Baseline		57.0000	11.0000	598.0000
CHR2mo		43.0000	6.0000	215.5000	CHR4mo		42.5000	7.5000	212.5000	CHR6mo		43.0000	4.0000	125.0000
CHR5mo		42.0000	6.0000	214.5000	CHR7mo		40.5000	6.0000	173.5000	CHR9mo		41.0000	4.0000	178.0000

* median.

**Table 3:** Statistical analysis of subgrouped patients (A,B,C) according to JAK2V617F VAF values and MPN-SAF TSS pruritus score at baseline (b) and durable CHR.

	A	VAF	Pruritus	JAK2/ Pruritus		B	VAF	Pruritus	JAK2/ Pruritus		C	VAF	Pruritus	JAK2/ Pruritus
Baseline		55	5.5	*p* 0.015 * p 0.004 **	Baseline		35	5	*p* 0.001 * p 0.002 **	Baseline		42	6	*p* 0.045 * p 0.015 **
CHR5mo		23	0, absent		CHR7mo		5	0, absent		CHR9mo		11	0, absent	

* Pearson, ** Spearman.

## Data Availability

The datasets presented in this article are not available due to technical limitations.
